# Activation of TAF9 *via* Danshensu-Induced Upregulation of HDAC1 Expression Alleviates Non-alcoholic Fatty Liver Disease

**DOI:** 10.3389/fphar.2021.775528

**Published:** 2021-12-02

**Authors:** Ruiwen Wang, Zhecheng Wang, Ruimin Sun, Rong Fu, Yu Sun, Meiyang Zhu, Yunfei Geng, Dongyan Gao, Xiaofeng Tian, Yan Zhao, Jihong Yao

**Affiliations:** ^1^ Department of Pharmacology, Dalian Medical University, Dalian, China; ^2^ Department of General Surgery, The Second Affiliated Hospital of Dalian Medical University, Dalian, China

**Keywords:** NAFLD, TAF9, HDAC1, Danshensu, FATTY ACID *β*-OXIDATION, LD accumulation

## Abstract

Fatty acid *β*-oxidation is an essential pathogenic mechanism in nonalcoholic fatty liver disease (NAFLD), and TATA-box binding protein associated factor 9 (TAF9) has been reported to be involved in the regulation of fatty acid *β*-oxidation. However, the function of TAF9 in NAFLD, as well as the mechanism by which TAF9 is regulated, remains unclear. In this study, we aimed to investigate the signaling mechanism underlying the involvement of TAF9 in NAFLD and the protective effect of the natural phenolic compound Danshensu (DSS) against NAFLD via the HDAC1/TAF9 pathway. An *in vivo* model of high-fat diet (HFD)-induced NAFLD and a palmitic acid (PA)-treated AML-12 cell model were developed. Pharmacological treatment with DSS significantly increased fatty acid *β*-oxidation and reduced lipid droplet (LD) accumulation in NAFLD. TAF9 overexpression had the same effects on these processes both *in vivo* and *in vitro*. Interestingly, the protective effect of DSS was markedly blocked by TAF9 knockdown. Mechanistically, TAF9 was shown to be deacetylated by HDAC1, which regulates the capacity of TAF9 to mediate fatty acid *β*-oxidation and LD accumulation during NAFLD. In conclusion, TAF9 is a key regulator in the treatment of NAFLD that acts by increasing fatty acid *β*-oxidation and reducing LD accumulation, and DSS confers protection against NAFLD through the HDAC1/TAF9 pathway.

## Introduction

Nonalcoholic fatty liver disease (NAFLD), a chronic disease which affects over 25% of the global population (Younossi et al., 2016). The clinical spectrum of NAFLD includes many kinds of diseases: steatohepatitis, fibrosis, and even cirrhosis and hepatocellular carcinoma (Estes et al., 2018). The pathogenesis of NAFLD is multifactorial, and the dominant feature of NAFLD is the accumulation of abundant fatty acids, which are mainly stored in lipid droplets (LDs) (Farrell et al., 2006). Previous study has demonstrated that fatty acid *β*-oxidation, which is one of the most important metabolic pathways of fatty acids (Li et al., 2014), plays an essential role in metabolic diseases progression such as insulin resistance, type 2 diabetes, atherosclerosis, dyslipidemia and NAFLD (Rogge et al., 2009). Thus, exploring a target of NAFLD to increase fatty acid *β*-oxidation may provide therapeutic strategies for NAFLD.

Transcription factor IID (TFIID), which is a complex for preinitiation complex assembly, can be recruited to the core promoter element (DPE) (Bieniossek et al., 2013). TAF9, a small TATA-binding protein (TBP)-associated factor (TAF), is a component of the complex TFIID of the DPE (Zeng et al., 2018). TAF9 directly regulates the transcription of target genes through binding to the DPE (Soldatov et al., 1999). A previous study showed that loss of function of TAF9 leads to an increase in LD size in the fat body of Drosophila larval (Fan et al., 2017). TAF9 can affect the expression of acyl-CoA oxidase 1 (ACOX1), which is an important enzyme in fatty acid *β*-oxidation (Yang et al., 2014), to regulate phospholipids fatty acid composition. Therefore, TAF9 is probably related to fatty acid *β*-oxidation. However, whether TAF9 participates in the progression of NAFLD and the mechanism by which TAF9 is regulated remains unclear.

A recent study showed that TAF9 acetylation regulates TFIID recruitment (Jian et al., 2017). Thus, the regulation of TAF9 acetylation may be associated with its function and activity. Histone deacetylase 1 (HDAC1) can deacetylate nonhistone and histones proteins and plays important roles in regulating the progression of diseases (Yang et al., 2007). A previous study demonstrated that HDAC1/2 bind to the fatty acid synthase promoter and induce deacetylation of H3K9 and H3K27 (Liu et al., 2018), suggesting that HDAC1 may participate in the progression of fatty liver disease. Furthermore, recent research has revealed that HDAC1 can regulate TAF9 deacetylation in human erythroleukemia K562 cells. Using HDAC inhibitor can increase the level of TAF9 acetylation (Jian et al., 2017). Above all, we hypothesize that HDAC1 may deacetylate TAF9 and increase fatty acid *β*-oxidation, thereby alleviating NAFLD.

*Salviae miltiorrhizae* Radix et Rhizoma (Danshen), a Chinese traditional medicinal herb, is edible and low toxicity (Cao et al., 2019). Danshensu (DSS, *β*-3, 4-dihydroxyphenyllactic acid) is an active water-soluble monomer of Danshen. Previous studies have demonstrated that DSS exerts a lot of pharmacological activities, such as activities against cancer, cardiovascular disease and liver injury (Ren et al., 2010; Chen et al., 2011; Cao et al., 2017). DSS may also contribute to hepatoprotective effects against ALD and NAFLD via pharmacological network analysis (Hong et al., 2017) and that DSS Bingpian Zhi (DBZ) prevents high-fat diet (HFD)-related metabolic syndrome (Xu et al., 2017). However, whether DSS can alleviate NAFLD, as well as the underlying mechanism, remains unclear.

In this study, our aims were as follows: 1) to explore whether TAF9 can be involved in NAFLD; 2) to explore the role of deacetylation of TAF9 by HDAC1 in NAFLD; and 3) to test whether DSS can alleviate NAFLD through the HDAC1/TAF9 signaling pathway.

## Materials and Methods

### Animals and Treatments

We purchased male Sprague-Dawley rats (200 ± 20 g) from the Dalian Medical University Animal Center (Dalian, China). We purchased DSS (98% purity) from MUST Biotechnology Co., Ltd. (Chengdu, China). Forty rats were divided into the five groups randomly of eight animals each after acclimatization for 1 week: 1) the normal diet (ND) group, 2) the ND + DSS (40 mg/kg) group, 3) the HFD group, 4) the HFD + DSS (20 mg/kg) group, and 5) the HFD + DSS (40 mg/kg) group. We gavaged the rats with DSS and olive oil every day and fed a HFD (8.3% yolk, 7% lard, 2% cholesterol, 16.7% sucrose, and 66% standard diet) or an irradiated standard ND in a place free of pathogens. This HFD contained 4.66 kcal/g, and the energy composition of HFD were as follows: 31.59% from fat, 16.68% from protein, and 51.73% from carbohydrate (Li et al., 2011; Zeng et al., 2015). 8 weeks later, animals were euthanized. We refered to Use Committee of Dalian Medical University and Institutional Animal Care guidelines to conduct the procedures, and the procedures were approved by Dalian Medical University Institutional Ethics Committee.

Male C57BL/6 mice (20 ± 20 g) were purchased from Dalian Medical University Animal Center (Dalian, China). They were fed a ND or HFD for 8 weeks. We constructed a lentiviral vector to overexpress TAF9 in mice. Through the tail vein, the mice were injected with lentivirus-scramble (1 × 10^9^ viral particles/mouse) or lentivirus-TAF9-shRNA (GenePharma, Shanghai, China) 1 week before ND or HFD feeding. EF-1α promoter was used to drive the expression of TAF9 in the lentivirus construct. And the TAF9 cDNA in the lentiviral vector was species of mouse. The lentiviruses were systemically expressed in every organ in the body. We referred to Use Committee of Dalian Medical University and Institutional Animal Care guidelines to conduct the procedures, and the procedures were approved by Dalian Medical University Institutional Ethics Committee.

### Cell Culture and Treatment

AML-12 cell line was cultured in a 37°C and 5% CO_2_ humidified incubator, and in DMEM/F12 (1:1) with transferrin (0.005 mg/ml), insulin (0.005 mg/ml), 10% (v/v) FBS, selenium (0.005 mg/ml), and dexamethasone (40 ng/ml) (Gibco, CA, United States). The cell line was purchased from ATCC (RRID: CVCL_0140, Rockefeller, United States; Cat #CRL-2254). AML-12 cells were used to establish the cellular fat accumulation cell model via treated with 400 μM palmitic acid (PA) (Sigma, No. P-0500) (Peck et al., 2010). After incubation with 5 μM Entinostat (Selleck, S1053) for 72 h and/or 10 μM DSS (Jingfeng Pharmaceutical Co., Ltd., Chengdu, China) for 6 h, the cells were stimulated with PA for 24 h.

### Histological Analysis

4% paraformaldehyde was used to collect the isolated liver lobes. And then, we embedded the liver in paraffin for preparing frozen sections. We used the liver sections to perform Oil Red O staining and hematoxylin-eosin (H&E) experiments.

### Immunohistochemical Staining

We performed immunohistochemistry (IHC) of TAF9 on paraffin sections (4 μm). TAF9 (Proteintech, Wuhan, China) antibody was used to incubate the liver sections overnight. And the sections were visualized with hematoxylin and DAB. We used ImageJ software to quantify positive cells number.

### Biochemical Assays

Assay kits (Jiancheng Corp., Nanjing, China) was used to measure Aspartate aminotransferase (AST), alanine aminotransferase (ALT), total cholesterol (TC) and triglyceride (TG) levels.

### Transmission Electron Microscopy

LD volume was determined using transmission electron microscopy (TEM). We fixed and embedded AML-12 cells and prepared 50 μm thick ultrathin sections. Then, we dehydrated these in an ethanol gradient, and then embedded, and stained with citrate and uranyl acetate. Finally, we used transmission electron microscope (JEOL, Peabody, MA) to obtain images.

### Nile Red Staining

We performed Nile red staining (Sigma, No. 19123) to detect intracellular lipid accumulation. We used fluorescence microscope to obtain the images.

### Cell Transfection

Plasmids of pcDNA, small interfering RNAs (siRNAs), or a negative control (GenePharma, Shanghai, China) were transfected into AML-12 cells for 48 h, which refered to the protocol of manufacturer using Lipofectamine 3000 (Invitrogen, Carlsbad, CA, United States). The plasmids and siRNAs were transfected for 48 h before PA exposure.

### Western Blotting

SDS-PAGE (8–12%) was performed to separate proteins. We used primary antibodies against the proteins to perform western blotting experiments: TAF9 (Proteintech, Wuhan, China), HDAC1 (Proteintech, Wuhan, China), CPT1A (Proteintech, Wuhan, China), ACOX1 (ABclonal Biotechnology, Wuhan, China), PPARα (ABclonal Biotechnology, Wuhan, China), ACSL1 (ABclonal Biotechnology, Wuhan, China), ADRP (Proteintech, Wuhan, China), and *β*-actin (Proteintech, Wuhan, China).

### RNA Isolation and qRT-PCR

We used TRIzol reagent (Invitrogen, Carlsbad, CA, United States) to extract the total RNA. We used PrimeScriptTM RT Reagent Kit (TaKaRa, Japan) to compound cDNA. SYBR Premix EX TaqTM ii (TaKaRa) was used to perform real-time PCR. The primers were shown in Supplementary Figure S6. We used the level of an internal control (β-actin) to normalize the PCR results.

### Immunoprecipitation and Coimmunoprecipitation Experiments

IP lysis buffer (1% Triton X-100, 150 mM NaCl and 20 mM Tris-HCl, pH 7.5) was used to extract total proteins. The equal amounts of an anti-TAF9 antibody and protein A/G magnetic beads (Bimake, Selleck Chemical, Houston, United States) was used to incubate the precleared lysates, which refered to the protocols of manufacturers (Zhao et al., 2018). The anti-TAF9, anti-HDAC1 or anti-acetyl lysine primary antibodies were used to immunablot the samples.

### Statistical Analysis

Student’s unpaired *t*-test (two-group comparisons) and one-way ANOVA (multigroup comparisons) were carried out with GraphPad Prism for statistical analyses. The results are expressed as the mean ± SD, and we considered statistical significant with *p* < 0.05.

## Results

### DSS Attenuates HFD-Induced NAFLD *in vivo*

**FIGURE 1:**
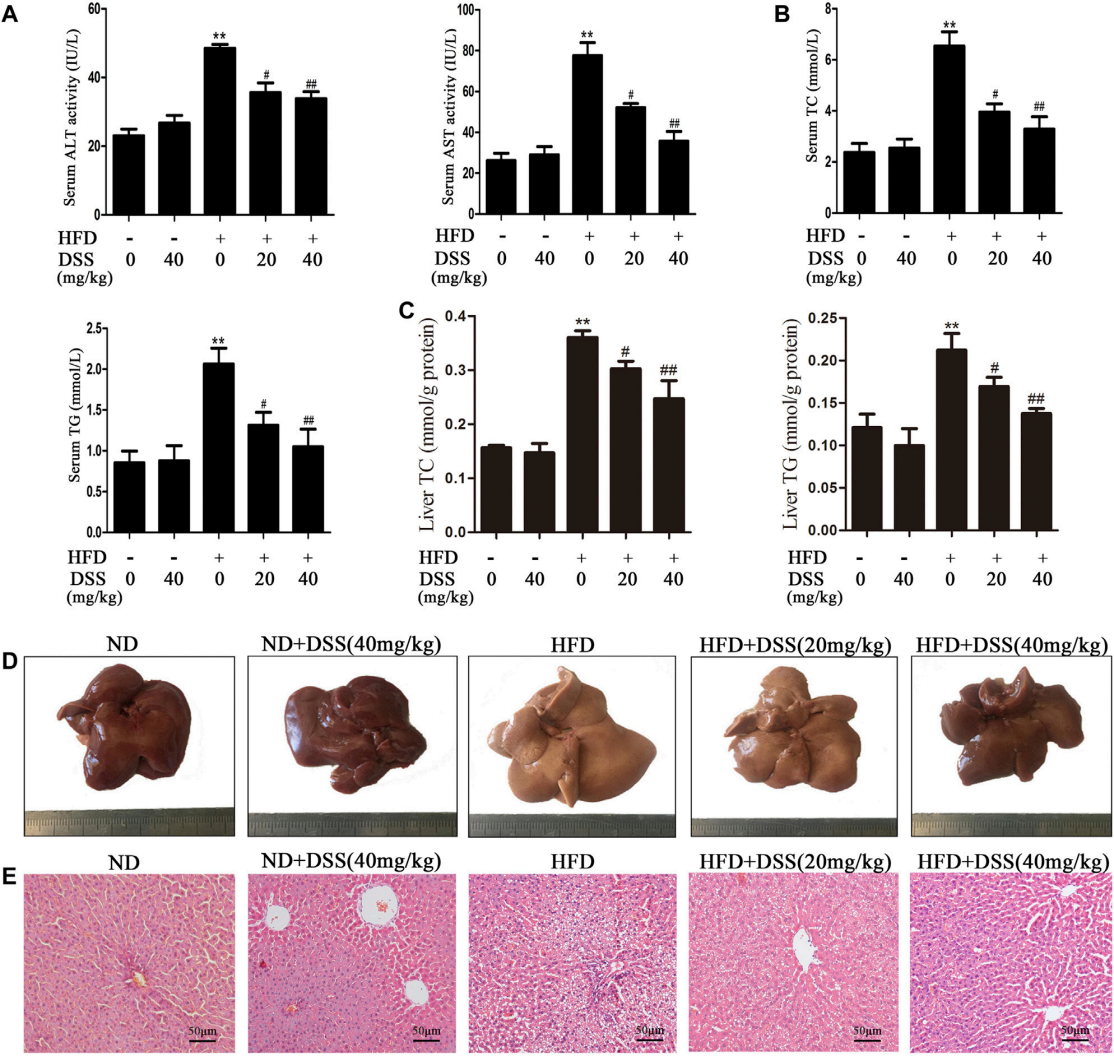
DSS alleviates HFD-induced NAFLD *in vivo*. Rats were fed either a ND (control) or a HFD and treated with or without DSS. **(A)** Serum levels of ALT and AST (*n* = 6). **(B)** Serum levels of TC and TG (*n* = 8). **(C)** Liver levels of TC and TG (*n* = 8). ^**^ *p* < 0.01 vs. the ND group; ^#^ *p* < 0.05, ^##^ *p* < 0.01 vs. the HFD group. **(D,E)** Representative morphological and H&E-stained images of liver sections from the following experimental groups: the ND, DSS (40 mg/kg), HFD, HFD + DSS (20 mg/kg) and HFD + DSS (40 mg/kg) groups. H&E-stained sections were photographed at 400× magnification.

Firstly, we determined whether DSS can protect HFD-induced NAFLD. Compared with the ND group, serum ALT, AST, TG, TC levels, liver TG, TC levels, body and liver weights were obviously increased in the HFD group, but DSS treatment clearly reversed these changes (Figures 1A–C; Supplementary Figures S1A,C). Moreover, liver sections histological and morphologic examinations revealed a lot of lipid vacuoles and a marked alteration in liver color in the HFD-fed rats hepatocytes. However, these changes can be attenuated by DSS treatment (Figures 1D,E). The results indicated that DSS attenuates NAFLD in rats.

### DSS Increases Fatty Acid *β*-oxidation and Attenuates LD Accumulation

**FIGURE 2:**
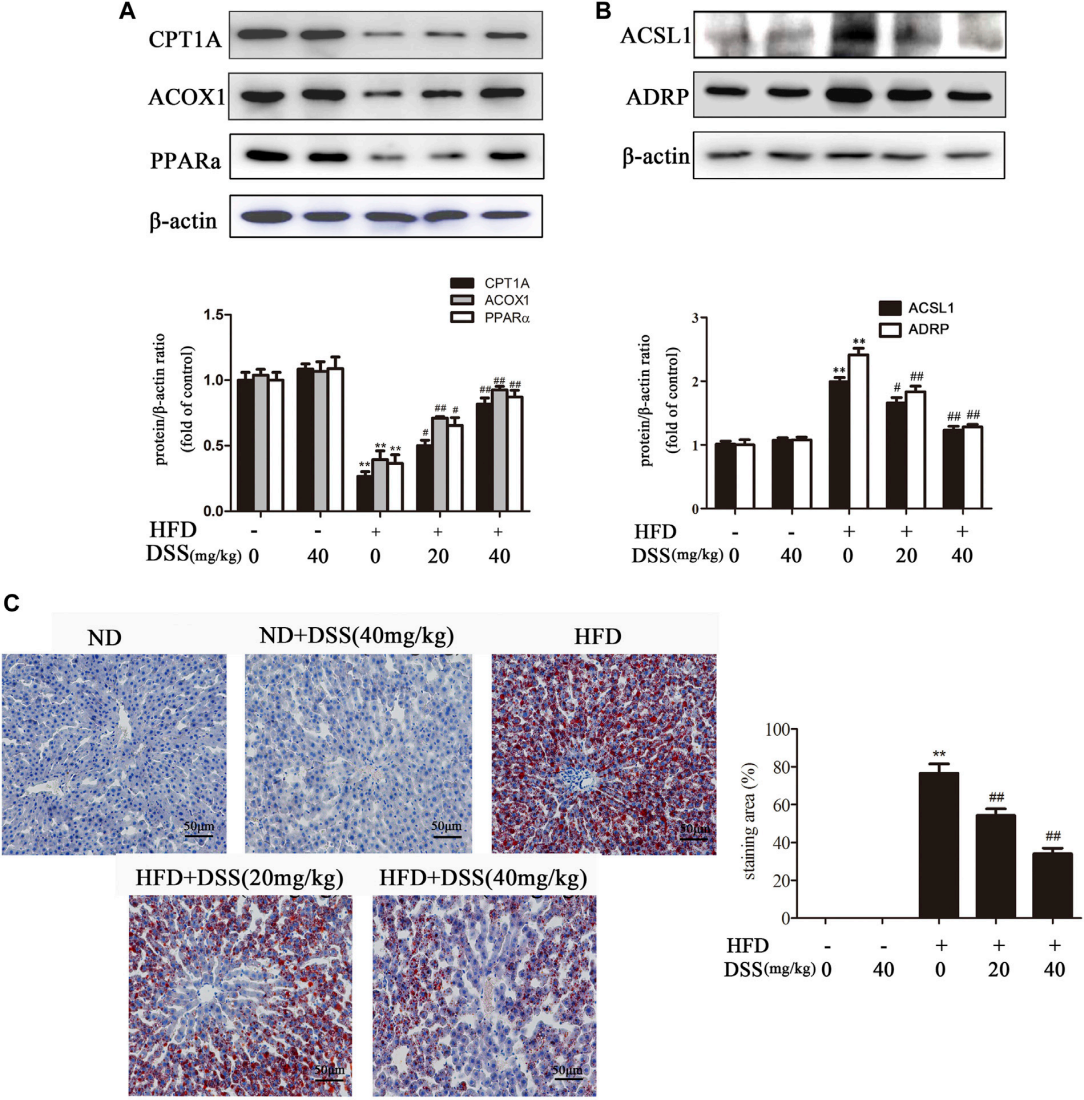
DSS increases fatty acid β-oxidation and attenuates LD accumulation in rats. **(A,B)** Western blot analysis of fatty acid *β*-oxidation-related genes (CPT1A, ACOX1 and PPARα) and LD accumulation-related genes (ACSL1 and ADRP) protein levels in the liver (*n* = 3). **(C)** Oil Red O-stained images of liver sections from the following experimental groups: the ND, DSS (40 mg/kg), HFD, HFD + DSS (20 mg/kg) and HFD + DSS (40 mg/kg) groups. Oil Red O-stained sections were photographed at 400× magnification. ^**^ *p* < 0.01 vs. the ND group; ^#^ *p* < 0.05, ^##^ *p* < 0.01 vs. the HFD group.

Fatty acid *β*-oxidation is a critical metabolic pathway in NAFLD. Blocking fatty acid *β*-oxidation can lead to liver LDs accumulation, which in turn results in NAFLD (Fabbrini et al., 2015; Martín-Pozuelo et al., 2015). Subsequently, we investigated the effect of DSS on fatty acid *β*-oxidation and LD accumulation both *in vivo* and *in vitro*. DSS treatment reversed the HFD-mediated downregulation of fatty acid *β*-oxidation-related genes (CPT1A, ACOX1 and PPARα) expression and upregulation of LD accumulation-related genes (ACSL1 and ADRP) expression (Figures 2A,B). Oil Red O staining experiment indicated that DSS treatment attenuated HFD-induced lipid accumulation in the liver (Figure 2C). These results demonstrated that DSS can protect NAFLD via promoting fatty acid *β*-oxidation and attenuates LD accumulation in rats.

**FIGURE 3:**
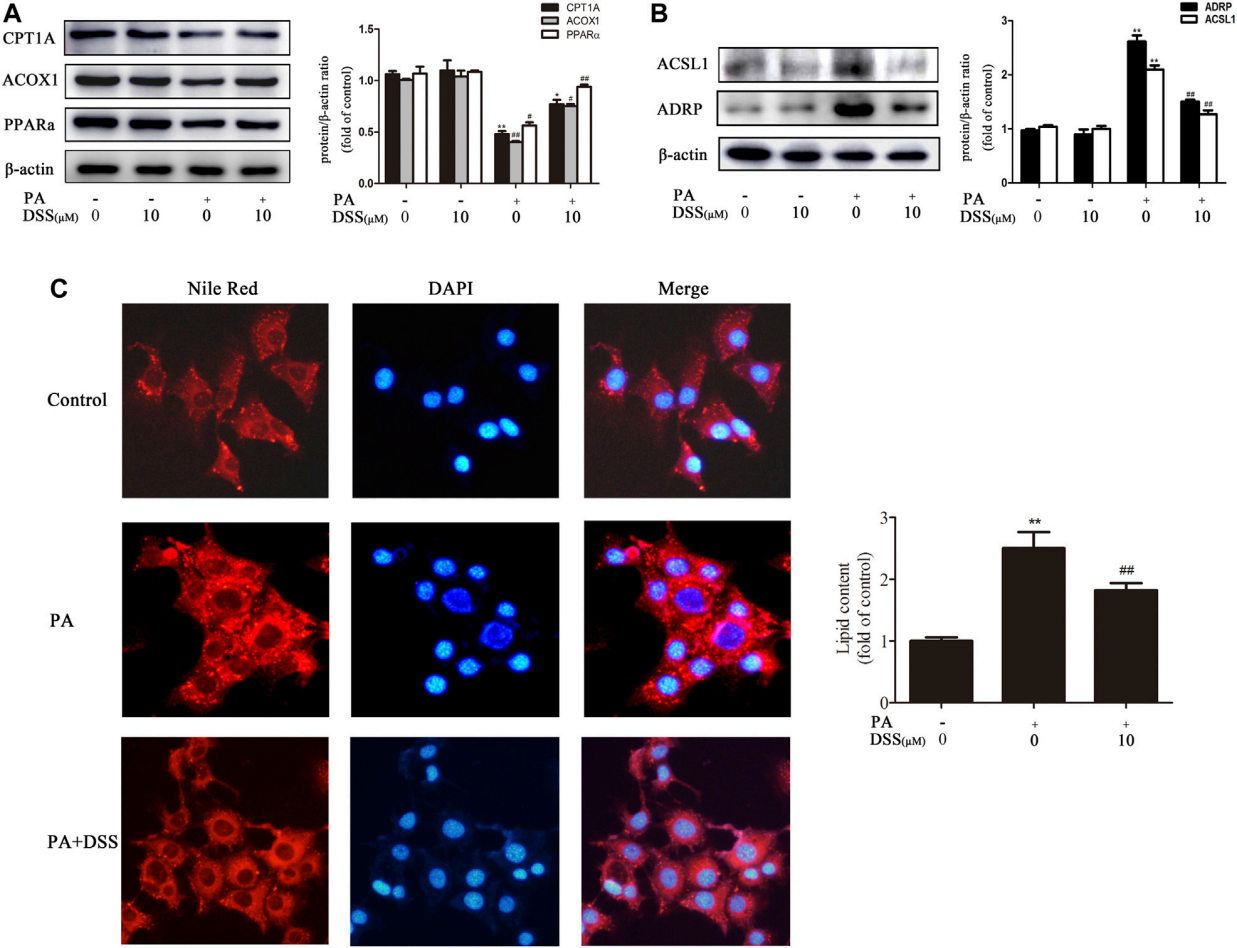
DSS promotes fatty acid β-oxidation and reduces LD accumulation *in vitro*. AML-12 cells were pretreated with DSS (10 μM) for 6 h before exposure to PA (0.4 mM) for another 24 h **(A,B)** Western blot analysis of fatty acid *β*-oxidation-related genes (CPT1A, ACOX1 and PPARα) and LD accumulation-related genes (ACSL1 and ADRP) protein levels in the cell lysates (*n* = 3). **(C)** Intracellular lipid accumulation was measured by Nile red staining (200×). ^*^ *p* < 0.05, ^**^ *p* < 0.01 vs. the control group; ^#^ *p* < 0.05, ^##^ *p* < 0.01 vs. the PA group.

AML-12 cells are immortalized mouse hepatocytes that have been well documented as cellular models of NAFLD (Li et al., 2019; Wu et al., 2020). Therefore, to mimic *in vivo* NAFLD progression, AML-12 cells were treated with DSS before PA exposure. DSS increased fatty acid *β*-oxidation and attenuated LD accumulation, as evidenced by increased CPT1A, ACOX1 and PPARα expression and decreased ACSL1 and ADRP expression (Figures 3A,B). Consistently, Nile red staining experiments indicated that LDs number was increased significantly in the PA group, and DSS treatment can abrogate this increase (Figure 3C). Above all, these data indicated that DSS can increase fatty acid *β*-oxidation and attenuates LD accumulation both *in vivo* and *in vitro*.

### DSS Promotes Fatty Acid *β*-oxidation and Reduces LD Accumulation Through TAF9 Activation

**FIGURE 4:**
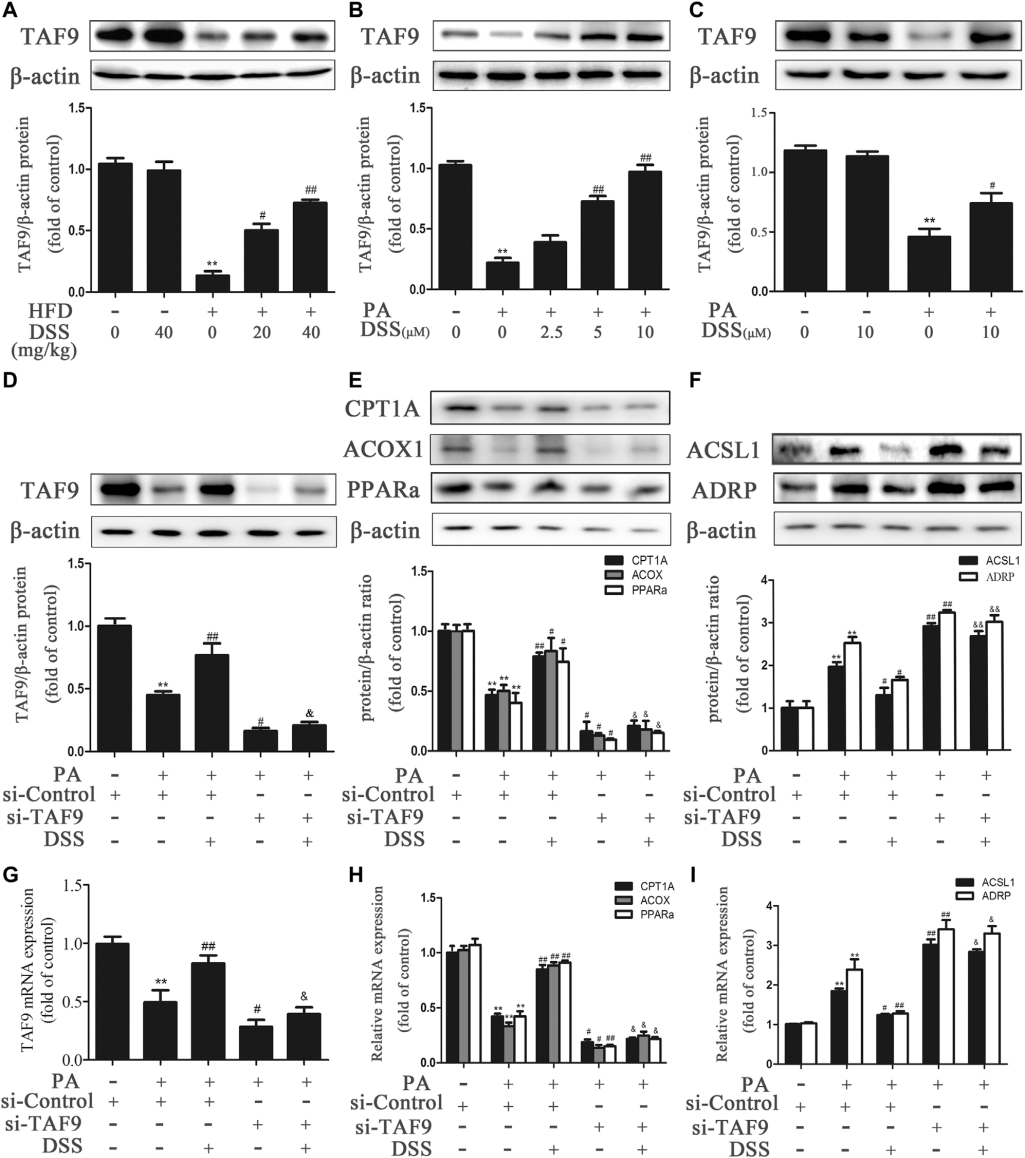
DSS promotes fatty acid β-oxidation and reduces LD accumulation through TAF9 activation. **(A)** Protein levels of TAF9 in the liver (*n* = 3). ^**^ *p* < 0.01 vs. the ND group; ^#^ *p* < 0.05, ^##^ *p* < 0.01 vs. the HFD group. **(B,C)** Protein levels of TAF9 in AML-12 cells (*n* = 3). ^**^ *p* < 0.01 vs. the control group; ^#^ *p* < 0.05, ^##^ *p* < 0.01 vs. the PA group. **(D–I)** AML-12 cells were transfected with a control siRNA or TAF9 siRNA for 48 h, and then the transfected cells were treated with DSS (10 μM) for 6 h before exposure to PA (0.4 mM) for another 24 h. Protein (*n* = 3) and mRNA (*n* = 6) levels of TAF9, fatty acid *β*-oxidation-related genes (CPT1A, ACOX1 and PPARα) and LD accumulation-related genes (ACSL1 and ADRP) in AML-12 cells. ^**^ *p* < 0.01 vs. the si-control group; ^#^ *p* < 0.05, ^##^ *p* < 0.01 vs. the si-control + PA group; ^&^ *p* < 0.05, ^&&^ *p* < 0.01 vs. the PA + DSS group.

We first measured the expression of TAF9 in HFD- and PA-induced NAFLD. As expected (Figures 4A–C), TAF9 expression was significantly decreased following HFD or PA treatment. However, DSS treatment abrogated the decrease in TAF9 expression both *in vivo* and *in vitro*. Subsequently, to explore the role of TAF9 in DSS-mediated fatty acid *β*-oxidation promotion and LD accumulation reduction, we transfected AML-12 cells with TAF9 siRNA and/or treated with DSS before exposured to PA. PA-treated AML-12 cells exhibited a decrease in TAF9 protein and mRNA expression (Figures 4D,G) accompanied by an significant decrease in fatty acid *β*-oxidation-related gene protein and mRNA expression (Figures 4E,H) and an increase in LD accumulation-related gene protein and mRNA expression (Figures 4F,I). However, DSS clearly upregulated TAF9 protein and mRNA expression and consequently reversed the decreases in fatty acid *β*-oxidation-related protein and mRNA levels and increases in LD accumulation-related protein and mRNA levels, but TAF9 knockdown can markly block these beneficial effects. Thus, these results suggested that DSS-mediated fatty acid *β*-oxidation promotion and LD accumulation reduction may be mediated by TAF9 activation.

### TAF9 Overexpression Attenuates HFD- and PA-Induced Fatty Acid *β*-oxidation Reduction and LD Accumulation

**FIGURE 5:**
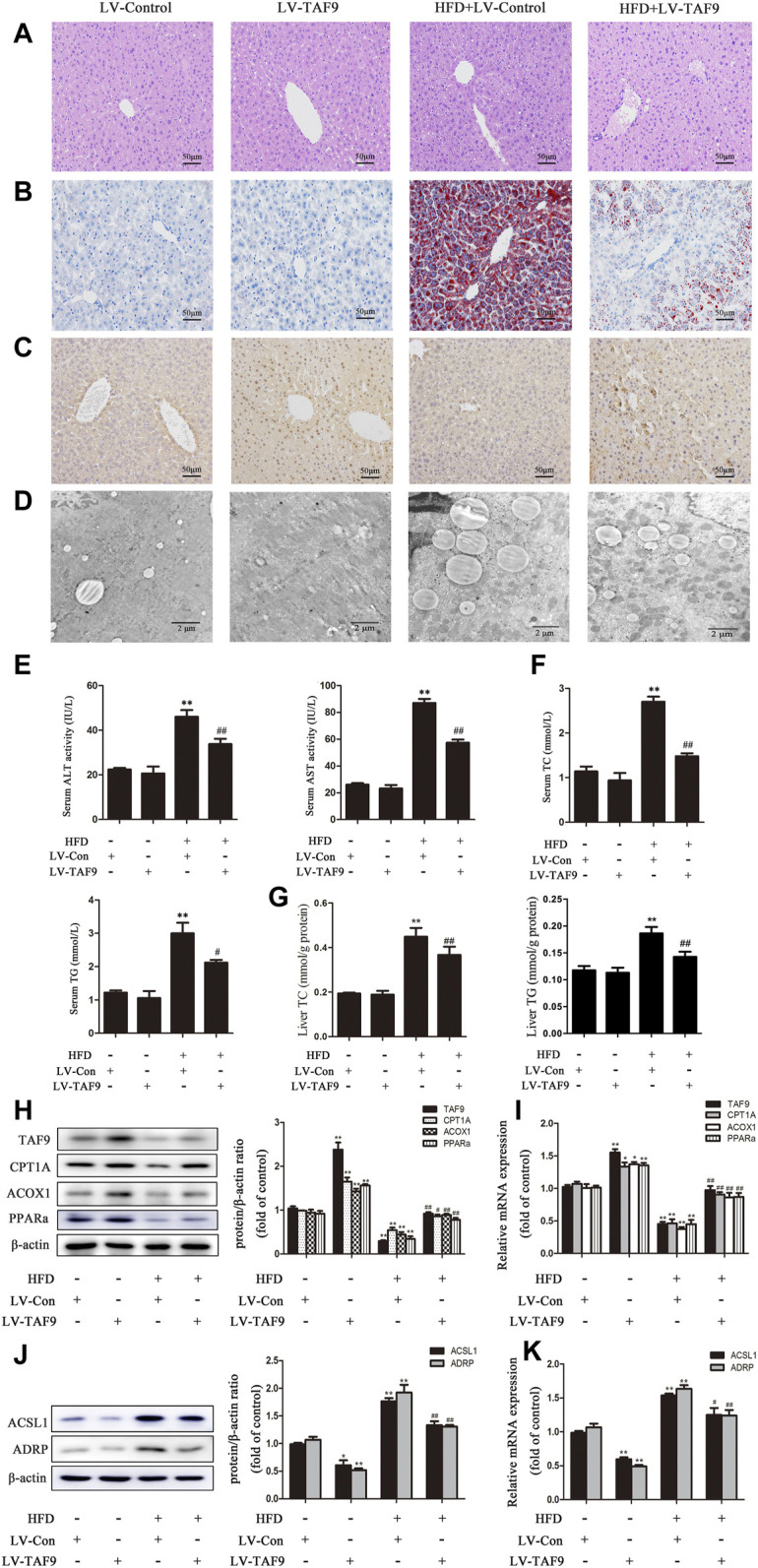
TAF9 overexpression attenuates HFD-induced fatty acid β-oxidation reduction and LD accumulation in mice. TAF9 overexpression was induced *via* lentiviruses delivered to C57BL/6 mice fed a HFD. **(A,B)** H&E-stained and Oil Red O-stained images of liver sections were taken at 400× magnification. **(C)** Images of TAF9 IHC staining of liver sections were taken at 400× magnification. **(D)** Electron microscopy images of liver sections were taken at 12000× magnification. **(E)** Serum levels of ALT and AST (*n* = 6). **(F)** Serum levels of TC and TG (*n* = 6). **(G)** Liver levels of TC and TG (*n* = 6). **(H–K)** Protein (*n* = 3) and mRNA (*n* = 6) levels of fatty acid *β*-oxidation-related genes (CPT1A, ACOX1 and PPARα) and LD accumulation-related genes (ACSL1 and ADRP) in the liver. ^*^ *p* < 0.05, ^**^ *p* < 0.01 vs. the LV-control group; ^#^ *p* < 0.05, ^##^ *p* < 0.01 vs. the HFD group.

To further determine the role of TAF9 in fatty acid *β*-oxidation and LD accumulation in NAFLD, we overexpressed TAF9 in mice using lentiviral transduction. According to H&E and Oil Red O staining, characteristic lipid accumulation was evident in the livers of HFD-fed mice, while TAF9 overexpression resulted in significantly less lipid accumulation (Figures 5A,B). We also observed the expression of TAF9 by immunohistochemistry and found that TAF9 was overexpressed in all types of cells in the lentivirus-infected liver (Figure 5C). TEM images showed that the volume of LDs increased significantly in mice fed a HFD. However, TAF9 overexpression significantly decreased the volume of LDs (Figure 5D). Consistently, TAF9 overexpression reversed HFD-induced increases in serum ALT, AST, TC and TG levels, liver TC and TG levels, body and liver weights in mice (Figures 5E–G; Supplementary Figures S1B,D). The results of a correlation analysis between TAF9 and ALT or AST is summarized in Supplementary Figures S2A,B. As expected, the level between TAF9 and ALT or AST was negatively correlated. Furthermore, in the context of NAFLD, lentiviral-mediated TAF9 overexpression changed the protein and mRNA levels of mediators of fatty acid *β*-oxidation and LD accumulation, as evidenced by increased CPT1A, ACOX1 and PPARα expression and decreased ACSL1 and ADRP expression (Figures 5H–K). We transfected AML-12 cells with pcDNA-TAF9 prior to PA treatment and found that TAF9 overexpression promoted fatty acid *β*-oxidation and attenuated LD accumulation *in vitro* (Supplementary Figure S3). These results demonstrated that overexpression of TAF9 attenuates HFD- and PA-induced fatty acid *β*-oxidation reduction and LD accumulation.

### DSS Promotes TAF9-Mediated Increases in Fatty Acid *β*-oxidation and Decreases in LD Accumulation Probably Through an HDAC1-dependent Mechanism

**FIGURE 6:**
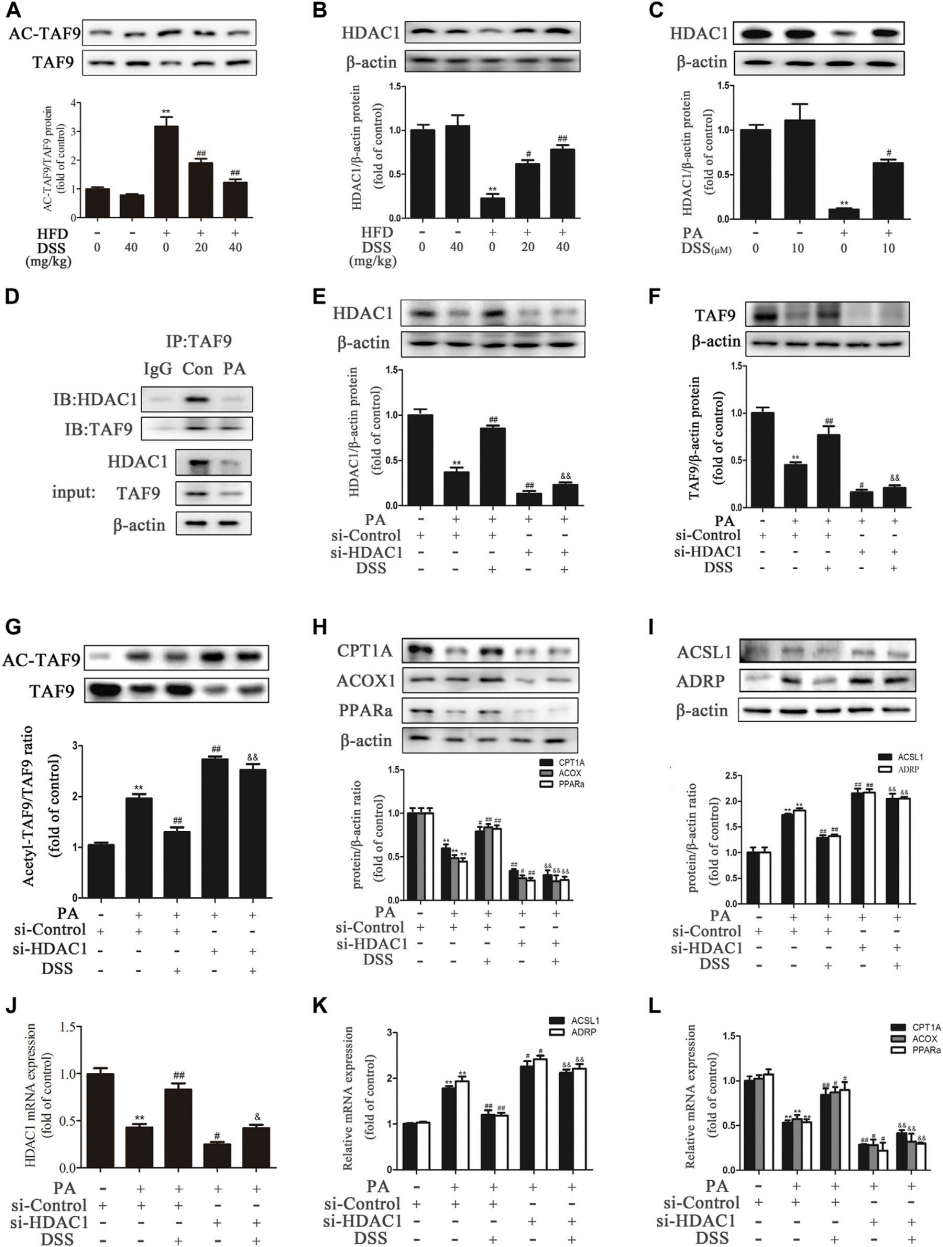
DSS promotes TAF9-mediated increases in fatty acid β-oxidation and decreases in LD accumulation, probably through an HDAC1-dependent mechanism. **(A,B)** Protein levels of TAF9, TAF9 acetylation and HDAC1 in the liver (*n* = 3). ^**^ *p* < 0.01 vs. the ND group; ^#^ *p* < 0.05, ^##^ *p* < 0.01 vs. the HFD group. **(C)** Protein level of HDAC1 in AML-12 cells (*n* = 3). ^**^ *p* < 0.01 vs. the control group; ^#^ *p* < 0.05vs. the PA group. **(D)** Representative immunoblot showing the interaction of HDAC1 with TAF9 in PA-treated AML-12 cells. The input represents the total protein extracts used for immunoprecipitation. IB, immunoblotting; IP, immunoprecipitation; IgG, negative control. **(E–L)** AML-12 cells were transfected with a control siRNA or HDAC1 siRNA for 48 h, and then the transfected cells were treated with DSS (10 μM) for 6 h before exposure to PA (0.4 mM) for another 24 h **(E–I)** Protein levels of HDAC1, TAF9, acetylated TAF9, fatty acid *β*-oxidation-related genes (CPT1A, ACOX1 and PPARα) and LD accumulation-related genes (ACSL1 and ADRP) in AML-12 cells (*n* = 3). **(J–L)** mRNA (*n* = 6) levels of HDAC1, fatty acid *β*-oxidation-related genes (CPT1A, ACOX1 and PPARα) and LD accumulation-related genes (ACSL1 and ADRP) in AML-12 cells. ^**^ *p* < 0.01 vs. the si-control group; ^#^ *p* < 0.05, ^##^ *p* < 0.01 vs. the si-control + PA group; ^&&^ *p* < 0.01 vs. the PA + DSS group.

It has been reported that acetylation of TAF9 can significantly decrease its activity and that HDAC1 can deacetylate TAF9 (Jian et al., 2017). Thus, we hypothesized that promoting deacetylation of TAF9 may increase its activity and attenuate NAFLD. Compared to ND feeding, HFD feeding increased the acetylation of TAF9, while DSS reduced the levels of acetylated TAF9 in rats, suggesting that DSS decreases the acetylation of TAF9 (Figure 6A). We thus explored the specific mechanism underlying the effect of DSS on TAF9. To evaluate whether HDAC1 contributes to DSS-mediated TAF9 deacetylation, we measured the expression of HDAC1 both *in vivo* and *in vitro*. DSS treatment reversed the HFD- and PA-stimulated decrease in HDAC1 expression (Figures 6B,C). We then explored the interaction between HDAC1 and TAF9 in the context of NAFLD using coIP experiments. In PA-treated AML-12 cells, HDAC1 was coimmunoprecipitated with TAF9, which demonstrated that there is a physical interaction between HDAC1 and TAF9 (Figure 6D). These data suggested that HDAC1 may contribute to DSS-mediated TAF9 deacetylation.

To further evaluate whether HDAC1 contributes to DSS-mediated increases in fatty acid *β*-oxidation and decreases in LD accumulation, AML-12 cells were transfected with HDAC1 siRNA or pretreated with DSS before PA exposure. DSS pretreatment reversed the PA-induced increases in acetyl-TAF9, ACSL1 and ADRP levels and the decreases in TAF9, CPT1A, ACOX1 and PPARα levels. However, HDAC1 knockdown can obviously block these effects (Figure 6E–L). The HDAC1 inhibitor (Entinostat) was also used to treat AML-12 cells only or in combination with DSS treatment in response to PA. As shown in Supplementary Figure S4, HDAC1 inhibitor treatment aggravated the PA-stimulated increases in ACSL1 and ADRP levels and the decreases in TAF9, CPT1A, ACOX1 and PPARα levels. Moreover, using the HDAC1 inhibitor in combination with DSS abolished the positive effect of DSS on fatty acid *β*-oxidation and LD accumulation-related gene expression. Furthermore, we also transfected AML12 cells with both HDAC1 and TAF9 siRNA, and detected the protein levels of HDAC1, TAF9, CPT1A, ACOX1, PPARα, ACSL1 and ADRP. As shown in Supplementary Figure S5, HDAC1 or TAF9 knockdown aggravated the PA-stimulated increases in ACSL1 and ADRP levels and the decreases in HDAC1, TAF9, CPT1A, ACOX1 and PPARα levels. Moreover, after transfected with HDAC1/TAF9 double knockdown, the protein levels of fatty acid *β*-oxidation and LD accumulation-related genes had no statistical difference compared with TAF9 knockdown group. The results indicated that the effect of HDAC1 on fatty acid *β*-oxidation and LD accumulation is at least partly via the regulation of TAF9. Taken together, these results suggested that DSS promotes TAF9-mediated increases in fatty acid *β*-oxidation and decreases in LD accumulation, probably through an HDAC1-dependent mechanism.

## Discussion

Currently, there is still a lack of effective treatment for NAFLD. Therefore, novel treatment targets and drugs are required. In this study, we first demonstrate that 1) TAF9 is an essential therapeutic target in the progression of NAFLD, 2) DSS can protect against HFD- and PA-induced fatty acid *β*-oxidation reduction and LD accumulation, and 3) DSS exerts a protective effect against NAFLD, possibly through the HDAC1/TAF9 pathway.

Fatty acids are mainly metabolized via mitochondrial *β*-oxidation, which is the major source of energy for the liver (Müller 1998; Wanders et al., 2011). When fatty acids are in excess supply or their processing is impaired, they may act as substrates for the production of lipotoxic substances, thereby stimulating endoplasmic reticulum (ER) stress and hepatocellular damage (Friedman et al., 2018; Li et al., 2018). Therefore, Clarifying the mechanisms of fatty acids (β-oxidation) in liver cells is critical to understand the metabolic basis of NAFLD. TAF9 is an evolutionarily conserved subunit of the SAGA and TFIID complexes (Fan et al., 2017). TAF9 affects the transcription of many genes in embryonic development (Jian et al., 2017). TAF9 also regulates Notch signaling in Drosophila (Xie et al., 2014). A recent study demonstrated that TAF9 affects genes transcription, including the fatty acid *β*-oxidation-related gene ACOX1. TAF9 can mediate peroxisomal ACOX1 expression to regulate the fatty acid composition of phospholipids (Fan et al., 2017). However, whether TAF9 can be involved in NAFLD remains unknown. In this study, we found that TAF9 expression was significantly downregulated both *in vivo* and *in vitro*. The level of TAF9 and ALT or AST in mice were negatively correlated. Furthermore, TAF9 overexpression markedly increased fatty acid *β*-oxidation and attenuated HFD- and PA-induced NAFLD, suggesting that TAF9-mediated fatty acid *β*-oxidation promotion plays crucial roles in NAFLD.

Hepatocytes can prevent lipotoxic damage by sequestering excess fatty acids and isolating them from the cytoplasm in response to defects in fatty acid oxidation or aberrant accumulation. In hepatocytes, primary fat reservoirs are located within dedicated storage organelles, cytoplasmic lipid droplets (LDs) (Byrne et al., 2015; Schulze et al., 2019). Dysregulation of hepatic lipid, which triggered by various factors, can lead to an imbalance between hepatic lipid anabolism and catabolism (Carr et al., 2016; Anete et al., 2018). Blocking lipid metabolism such as fatty acid oxidation can also lead to the accumulation of LDs (Fabbrini et al., 2015; Martín-Pozuelo et al., 2015). Thus, in this study, we determined the role of TAF9 in LD accumulation. We found that TAF9 overexpression significantly decreased the protein expression of LD accumulation-related genes (ACSL1 and ADRP) and attenuated the accumulation of LDs in HFD-induced NAFLD, as indicated by Oil Red O staining. Moreover, *in vitro*, TAF9 overexpression decreased the protein and mRNA levels of ACSL1 and ADRP and attenuated LD accumulation, as measured by Nile red staining. Taken together, the above findings show that TAF9 attenuates LD accumulation during NAFLD.

Post-translational modifications, such as lysine modifications, provide fine tuning control of intracellular protein function (Yamauchi et al., 2012; Black et al., 2012). According to bioinformatics predictions (http://plmd.biocuckoo.org/index.php) based on lysine modifications of TAF9, we found that TAF9 possesses acetylation, ubiquitination and sumoylation sites. However, only the acetylation sites (K5 and K244) of TAF9 were conserved among species. Consequently, we further explored the role of TAF9 acetylation in the progression of NAFLD. The function of HDACs is to remove acetyl groups from histone (and nonhistone protein) lysine residues (Draney et al., 2018). It has been reported that HDAC1 can regulate CDK8 in liver progenitor cells during regeneration (Ko et al., 2019). HDAC1 also leads to PTEN deacetylation in hepatocellular carcinoma (Qian et al., 2018). Therefore, HDAC1 is an important gene regulator in the liver through deacetylation of substrates. A previous study demonstrated that TAF9 and TFIID recruitment correlates with HDAC1 deacetylase activity and that TAF9 can be deacetylated by HDAC1 (Jian et al., 2017). In our study, we showed that HDAC1 expression was decreased in the PA-induced AML-12 cell model of NAFLD. DSS significantly increased HDAC1 expression and decreased the high level of acetylated TAF9. Moreover, HDAC1 directly binds and deacetylates TAF9, as demonstrated by coIP. The results of the HDAC1 and TAF9 double knockdown experiment indicated that the effect of HDAC1 on fatty acid *β*-oxidation and LD accumulation is at least partly due to the regulation of TAF9. More importantly, HDAC1 knockdown significantly blocked the DSS-mediated promotion of fatty acid *β*-oxidation and inhibition of LD accumulation. These data suggest that HDAC1 contributes to DSS-mediated TAF9 deacetylation and that HDAC1 may play an essential role in DSS-induced protection against NAFLD.

Chinese traditional herbs have been used in China for thousands of years. Preventing NAFLD using food- or herbal-derived natural products with an adverse reaction tolerance and broad efficacy is a promising approach to reducing morbidity (Li et al., 2020; Wang et al., 2020). To date, several active natural products, including carnosic acid (Shan et al., 2015), protocatechuic acid (Sun et al., 2020) and betulinic acid (Gu et al., 2019), have been shown to have potent effects against NAFLD. Therefore, it is reasonable to explore the efficacy of natural products for protecting NAFLD. DSS is a typical natural product, which derived from *S. miltiorrhizae* Radix et Rhizoma (Danshen). DSS has a variety of biological and pharmacological activities and may provide benefits to human health (Zhang et al., 2018; Bae et al., 2019; Zhang et al., 2019). Here, we found that DSS has the potential to prevent NAFLD. Mechanistically, DSS increased fatty acid *β*-oxidation and decreased LD accumulation via promotion of TAF9 expression in the context of NAFLD. Moreover, the promotion of TAF9 expression is closely associated with the upregulation of HDAC1 expression induced by DSS. Taken together, these data show that DSS has protective effects against NAFLD via the HDAC1/TAF9 signaling pathway. Furthermore, a previous study has explored the pharmacological network of DSS in ALD and NAFLD and found that PPARα, an important fatty acid *β*-oxidation-related gene in NAFLD, is one of the hepatoprotective targets of DSS (Hong et al., 2017). We also found that DSS could increase the PPARα protein level in NAFLD. Therefore, PPARα may also play an essential role in the treatment of NAFLD by DSS. Moreover, other key DSS-related enzymes or genes, such as PPARα in NAFLD could be explored in the further studies.

In summary, our study provides the first confirmation of the essential role of TAF9 in NAFLD. Furthermore, the results show that DSS promotes TAF9-mediated increases in fatty acid *β*-oxidation and decreases in LD accumulation during NAFLD, probably through an HDAC1-dependent mechanism. These findings highlight pharmacological activation of the HDAC1/TAF9 pathway as an attractive approach to alleviate NAFLD and identify DSS as a new candidate for NAFLD therapy.

## Data Availability

The original contributions presented in the study are included in the article/Supplementary Material, further inquiries can be directed to the corresponding authors.

## Abbreviations

ACOX1, acyl-CoA oxidase 1; ALT, alanine transaminase; AML-12, alpha mouse liver-12; AST, aspartate aminotransferase; DSS, Danshensu; HDAC1, histone deacetylase 1; HFD, high-fat diet; LD, lipid droplet; NAFLD, nonalcoholic fatty liver disease; PA, palmitic acid; qRT-PCR, quantificational reverse transcription-polymerase chain reaction; TAF9, TATA-binding protein-associated factor 9; TC, triglyceride; TG, total cholesterol.
