# Supplementary figures and images for: Activation of TAF9 via Danshensu-Induced Upregulation of HDAC1 Expression Alleviates Non-alcoholic Fatty Liver Disease
Source: Front Pharmacol. 2021 Dec 2;12:775528. doi: 10.3389/fphar.2021.775528 (PMC8678612; doi:10.3389/fphar.2021.775528)

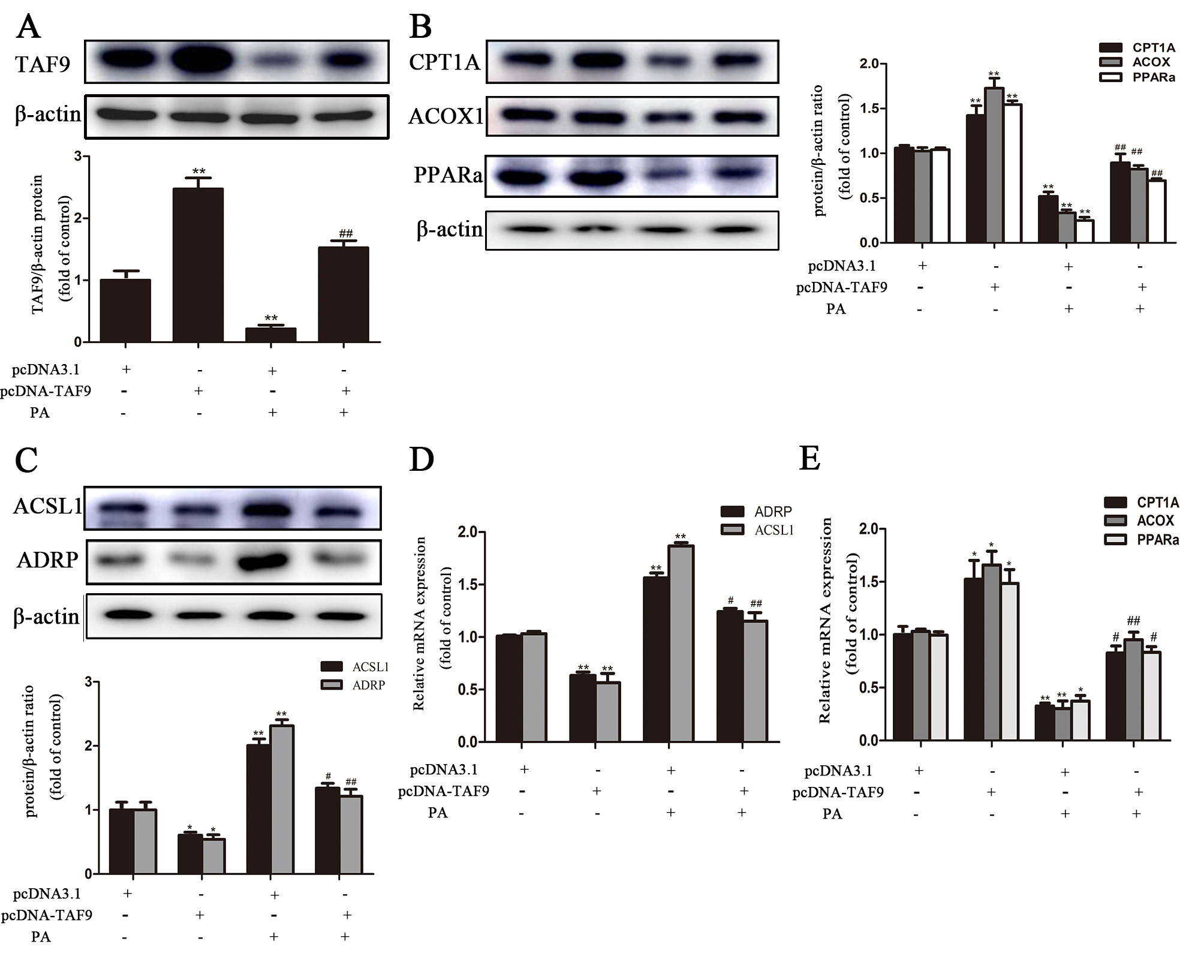

Supplement: Supplementary file 1 [file Image3.JPEG]

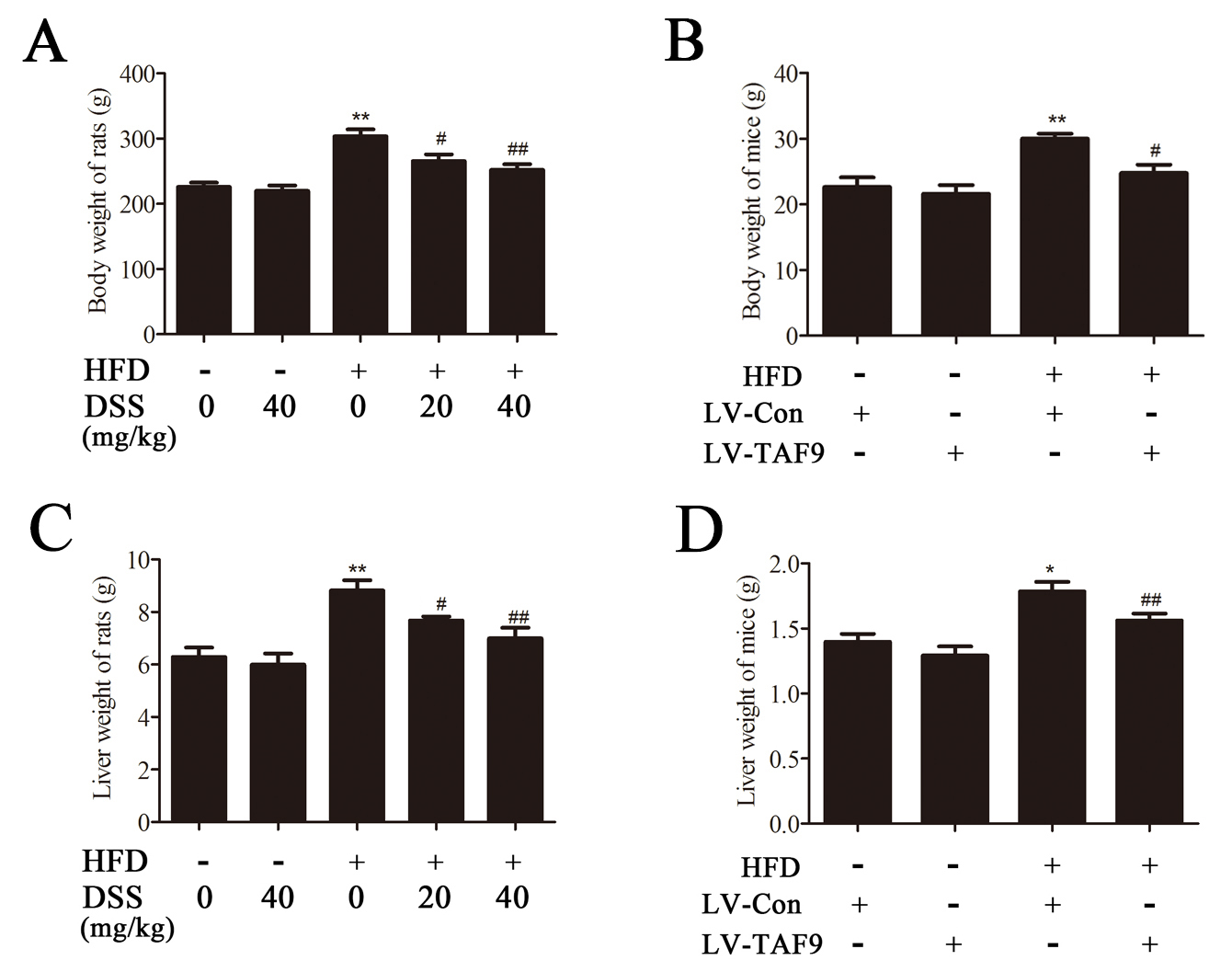

Supplement: Supplementary file 2 [file Image1.JPEG]

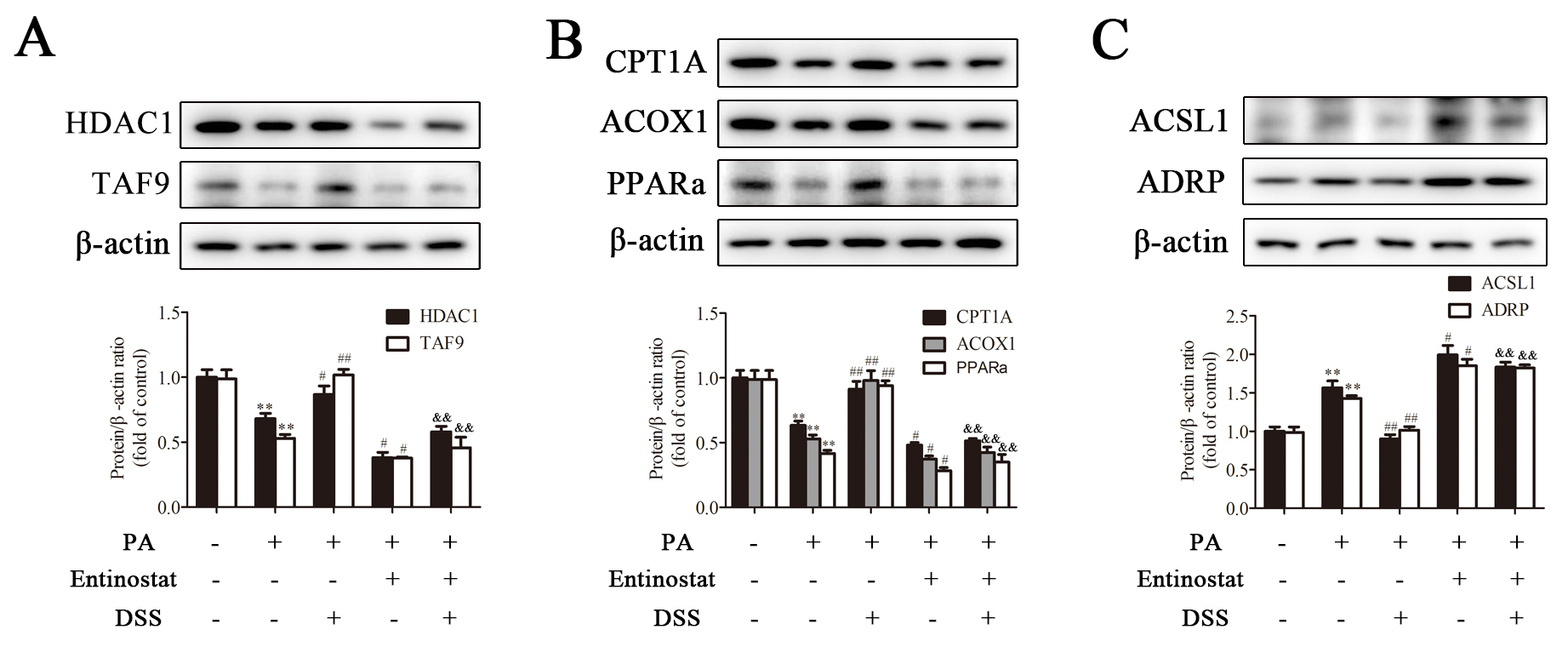

Supplement: Supplementary file 3 [file Image4.JPEG]

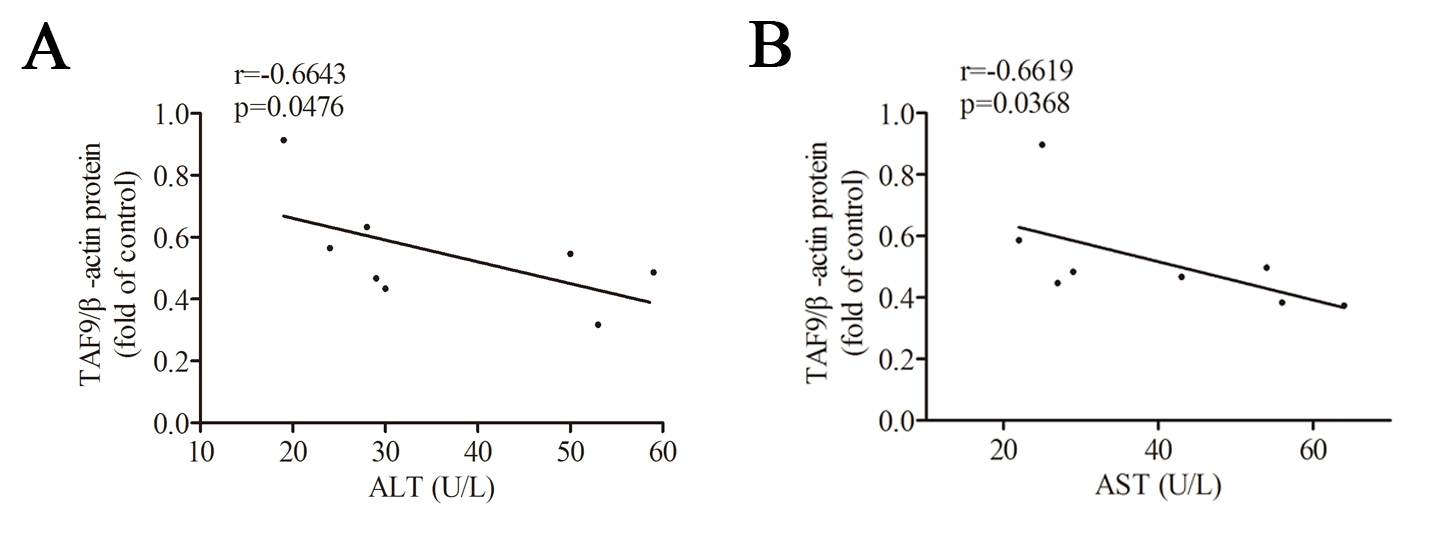

Supplement: Supplementary file 4 [file Image2.JPEG]

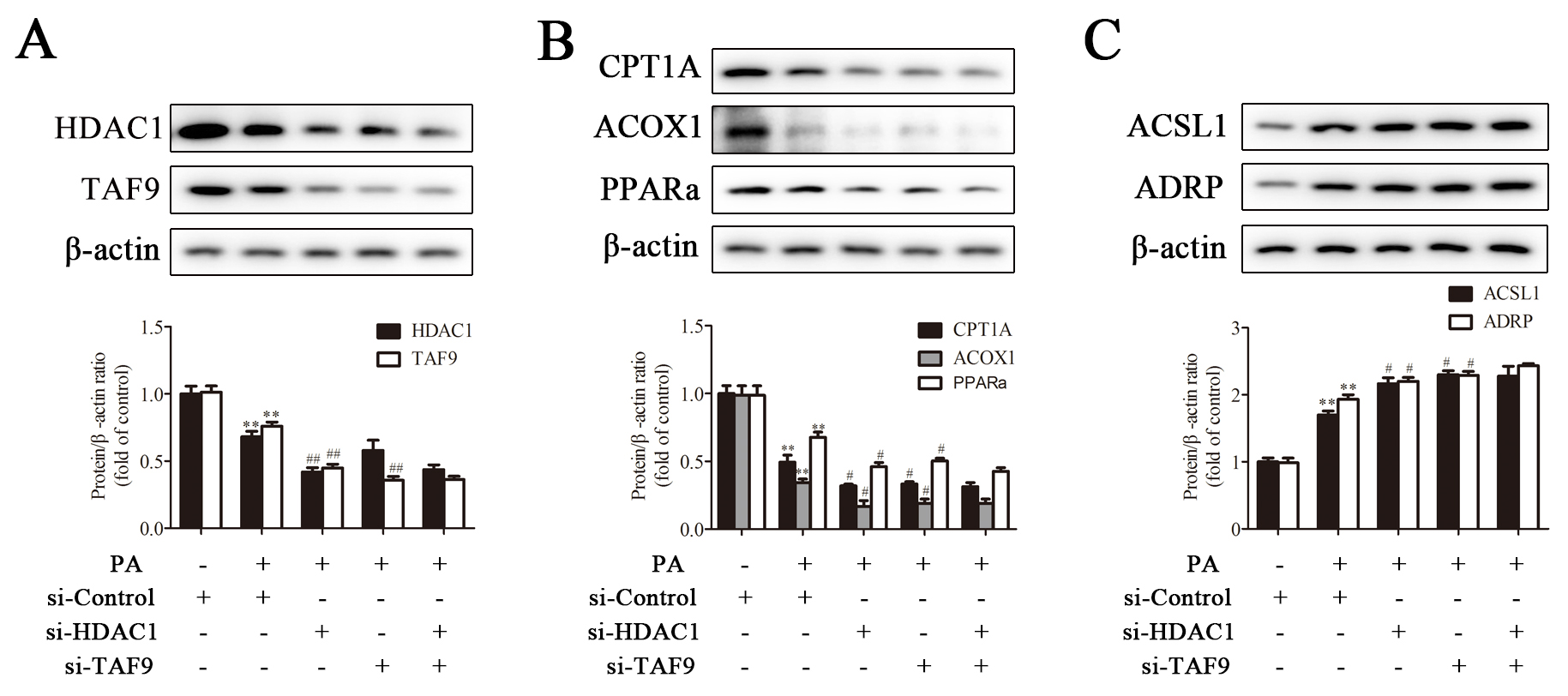

Supplement: Supplementary file 5 [file Image5.JPEG]

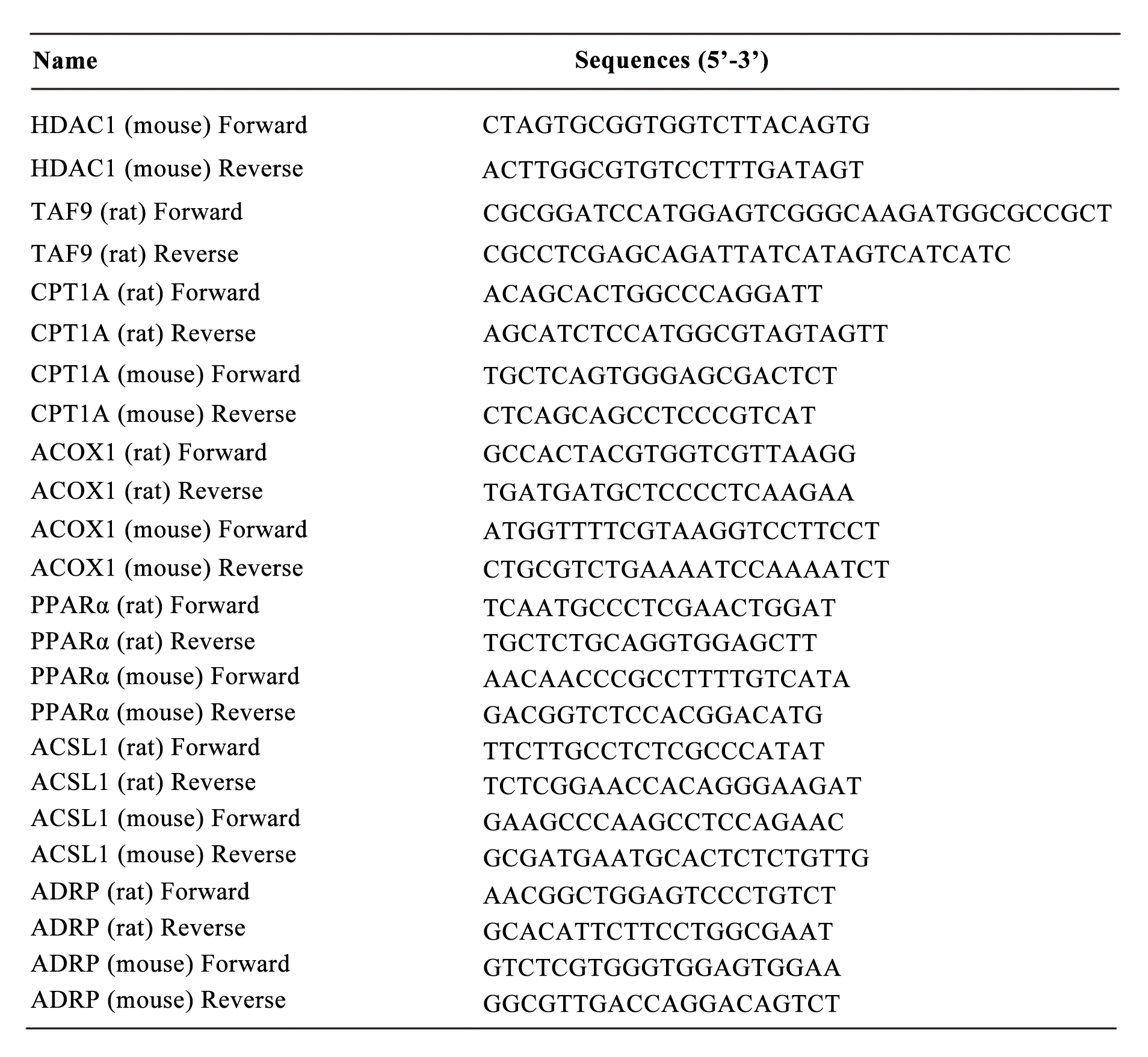

Supplement: Supplementary file 6 [file Image6.JPEG]
